# “Association between moderate renal insufficiency and cardiovascular events in a general population: Tehran lipid and glucose study”

**DOI:** 10.1186/1471-2369-13-59

**Published:** 2012-07-16

**Authors:** Farhad Hosseinpanah, Maryam Barzin, Hosein Aghayan Golkashani, Amir A Nassiri, Farhad Sheikholeslami, Fereidoun Azizi

**Affiliations:** 1 Obesity Research Center, Research Institute for Endocrine Science, Shahid Beheshti University of Medical Sciences, Tehran, Iran; 2 Prevention of Metabolic Disorders Research Center, Research Institute for Endocrine Science, Shahid Beheshti University of Medical Sciences, Tehran, Iran; 3 Endocrine Research Center, Research Institute for Endocrine Science, Shahid Beheshti University of Medical Sciences, Tehran, Iran; 4 Director, Obesity Research Center, Research Institute for Endocrine Sciences, Shahid Beheshti University of Medical Science, Tehran, Iran

**Keywords:** Chronic kidney disease, Cardiovascular disease, Body mass index, Glomerular filtration rate, General population

## Abstract

**Background:**

Chronic kidney disease(CKD) has been proposed as a risk factor for cardiovascular disease (CVD). There is conflicting evidence among community based studies regarding the association between CKD and CVD. Furthermore, in order to assess the possible interaction between CKD and BMI, we also examined the association between CKD and CVD, across different BMI categories.

**Methods:**

The risk of CVD events was evaluated in a large cohort of participants selected from the Tehran Lipid and Glucose Study. Participants(mean age, 47.4 years) free of previous CVD were followed up for 9.1 years. GFR ml/min per 1.73 m^2^ was estimated using the MDRD formula.

**Results:**

Of the 6,209 participants, 22.2%(1381) had CKD with eGFR ml/min per 1.73 m^2^ <60 at baseline. Almost all of them (99%) were in stage 3a. Moderate renal insufficiency only predicted CVD outcomes independently when we adjusted for age and sex. After further adjustment, the presence of moderate CKD lost its statistical significance to confer an independent increased risk of CVD events with a hazard ratio of: HR: 1.14, CI 95% 0.91-1.42. Furthermore, when participants were categorized according to CKD status and BMI groups, after further adjustment, no interaction was found(*P* = 0.2).

**Conclusion:**

CKD was not an independent risk factor for CVD events in a community-based study in a Tehranian population and the higher prevalence of CVD in subjects with mild to moderate renal insufficiency might be due to the co-occurrence of traditional CVD risk factors in this group.

## Background

Chronic kidney disease (CKD) is a major public health problem. Numerous studies on the epidemiology of CVD in end-stage renal disease (ESRD), have shown the significant burden of CVD [1-4]; also there has been heated debate regarding the reverse epidemiology phenomenon in this specific population, suggesting that increase in BMI (possibly due to increase in lean body mass) is associated with a higher survival rate [5]. However there is conflicting evidence among community based studies regarding the association between CKD and CVD as an independent risk factor.

Studies differ from each other in terms of population characteristics, method used to estimate glomerular filtration rate (GFR), outcome definition, adjustment for confounders and the time period of follow-up. Most studies conducted in general populations confirm the independent association [6-10]. There are also studies investigating surrogate CVD outcomes in people with early CKD showing that even moderate renal insufficiency can be considered an independent atherosclerosis risk factor in these individuals [11]. In contrast to these findings, there are community-based studies that suggest mild to moderate CKD does not independently predict CVD and the contribution is only due to the co-occurrence of traditional CVD risk factors [12,13].

Considering the lack of population-based cohort studies from the Middle East region addressing the role of CKD in predicting CVD and high prevalence of CKD in our population [14] we investigated the independent role of CKD in developing CVD in a large population of adult residents of Tehran during a median follow up of 9.1 years. Furthermore, most studies have investigated the possible interaction between CKD and BMI in ESRD patients on hemodialysis, raising the issue of “obesity paradox”, while fewer studies have addressed this research question in the general population. In order to assess the possible relationship between CKD and BMI, we also examined the association between CKD and CVD, across different BMI categories.

## Methods

### Study participants

The Tehran Lipid and Glucose Study (TLGS) is a prospective, population-based study being conducted to determine the risk factors for non-communicable diseases among a representative Tehran urban population [15]. In the TLGS, 15,005 subjects aged ≥3 years were selected by a multistage cluster random sampling method; of these, 8,071 participants aged ≥30 years were evaluated in the cross-sectional phase of the TLGS (February 1999 to August 2001). After the exclusion of those with prevalent CVD (n = 521) and underweight subjects (BMI <18.5 kg/m2; n = 106), those with missing anthropometric or biochemical data (n = 452) were also excluded, leaving 6992 participants with complete data; of these, 6,209 participants (88.9%) were followed until March 2009, with a median of 9.1 years (39448 person years). The ethics committee of the Research Institute for Endocrine Sciences approved this study, and informed written consent was obtained from all subjects.

### Baseline survey

At baseline, subjects were interviewed by trained interviewers using pretested questionnaires. Thereafter, demographic data collection, anthropometric examinations, medical history of CVD, medication use and smoking habit were undertaken by trained general physicians. Weight was measured while the subjects were minimally clothed without shoes using digital scales and recorded to the nearest 100 g. Height was measured in a standing position, without shoes, using a tape measure while the shoulders were in a normal position. BMI was calculated as weight in kilograms, divided by height in meters squared. Systolic and diastolic blood pressure were measured twice in a seated position after a 15-minute rest period using a standard mercury sphygmomanometer. Blood samples were taken after a 12 to 14-hour overnight fast. All blood analyses were done at the TLGS research laboratory on the day of blood collection [15]. Plasma total cholesterol (TC) and triglyceride (TG) levels were measured using enzymatic colorimetric kits (Pars Azmon Inc.); serum creatinine (mg/dl) were measured according to the standard colorimetric Jaffe_Kinetic reaction method (Pars Azmon Inc., Iran).

### Follow-up survey

Details of the collection of CVD outcome data have been published elsewhere [16]. Briefly, a trained nurse asked all participants of TLGS for any medical event by telephone call annually. Then, if any medical event had occurred a trained physician collected complementary data regarding that event during a home visit and by acquisition of data from medical files. An outcome committee consisting of an internist, endocrinologist, cardiologist, epidemiologist, and other experts evaluated the collected data to assign a specific outcome for every event. Coronary heart disease included cases of definite myocardial infarction (diagnostic electrocardiographic results and biomarkers), probable myocardial infarction (positive electrocardiographic findings plus cardiac symptoms or signs plus missing biomarkers or positive electrocardiographic findings plus equivocal biomarkers), angiographically proved coronary heart disease, and coronary heart disease death. CVD was defined as any coronary heart disease event, stroke (a new neurological deficit that lasted ≥24 hours), or CVD death.

### Definitions

Hypertension was defined as SBP/DBP ≥ 140/90 mm-Hg or patients taking antihypertensive agents. Hyper TG was defined as serum TG of ≥ 150 mg/dl and hyper TC of ≥ 240 mg/dl and both definitions also included subjects taking lipid lowering medications [17]. Smoking was defined as participants who smoked cigarettes daily or occasionally or participants who never smoked before. Diabetes was defined according to the criteria of the American Diabetes Association (ADA) as fasting plasma glucose ≥ 126 mg/dl or 2-h post-load ≥ 200 mg/dl or current therapy for a definite diagnosis of diabetes [18]. Family history of premature coronary artery disease (CAD) was defined as previous diagnoses of CAD in first-degree female relatives aged < 65 years or in first-degree male relatives aged < 55 years [19].

We also used the Modification of Diet in Renal Disease (MDRD) equation formula and GFR was expressed in ml/min per 1.73 m^2^ by multiplying each estimated GFR by 1.73/body surface area (m^2^) [20]. The abbreviated MDRD study equation is as follows:

GFR = 186 × (Serum creatinine)^-1.154^ × (Age)^-0.203^ × (0.742 if female)

Patients were classified as defined by national Kidney Foundation based on their eGFR levels [21]; subjects with 60 ml/min per 1.73 m^2^ and higher as not having CKD; subjects with 45 to 59 ml/min per 1.73 m^2^ (stage 3a); those with 30 to 44 ml/min per 1.73 m^2^ (stage 3b); with 15 to 29 ml/min per 1.73 m^2^ (stage 4) and finally those with less than 15 ml/min per 1.73 m^2^ as stage 5. For sake of simplicity in our analysis the participants were classified into the 2 groups: eGFR ≥60 ml/min per 1.73 m^2^ and eGFR <60 ml/min per 1.73 m^2^, the latter group being defined as CKD. Moreover, participants were classified into 3 categories according to BMI: Normal weight (18.5-24.9 kg/m^2^), Overweight (BMI 25–29.9 kg/m^2^) and Obese (≥30 kg/m^2^). In addition, the subjects were also classified into 6 groups based on CKD and BMI: 1- Normal weight without CKD, 2- Overweight without CKD, 3- Obese and without CKD 4- Normal weight with CKD, 5- Overweight with CKD and 6- Obese with CKD.

### Statistical methods

All continuous data with normal distribution are expressed as mean ± SD and skewed parameters as median, interquartile 25–75% (IQ25–75), and categorical variables are expressed as percentage. Difference for continuous variables was assessed using the *t*-test, whereas difference for categorical variables was assessed with the Chi Square Test.

Participants free of CVD at baseline were followed until the occurrence of a new ischemic cardiovascular event (the exact date of which was considered as the date of the end point event) or death or loss to follow-up, in which case the date of the last patient visit or the date of death due to a non-CVD event were considered as censoring. In the regression model, subjects were stratified into 6 categories according to BMI and CKD status, considering the group with normal weight and without CKD as the reference category. We calculated incidence rates and adjusted hazard ratios of CVD events according to BMI groups, in the presence or the absence of CKD. The person time for each participant was calculated from the beginning of the study to the date of CVD event or the end of the study, whichever came first. Incidence rates of CVD were obtained by dividing the number of cases by person-years in subgroup of BMI groups. Kaplan-Meier survival analysis was used to compare survival times among patients with CKD versus those without CKD. The log-rank statistic was used to test for differences among groups. Using Cox proportional- hazards models, relative risks were computed as the incidence rate in subgroups of BMI, divided by the incidence rate in the reference category. We tested for interaction between BMI categories and CKD status in development of CVD. The initial model was adjusted for age (years) and gender sex (reference: male). In the second multivariate model, we further adjusted for current smoking, family history of premature CAD, DM, high blood pressure, hyper TG and hyper TC. Stata version 10 was used for data analysis, *P* values <0.05 being considered statistically significant.

## Results

**Figure 1:**
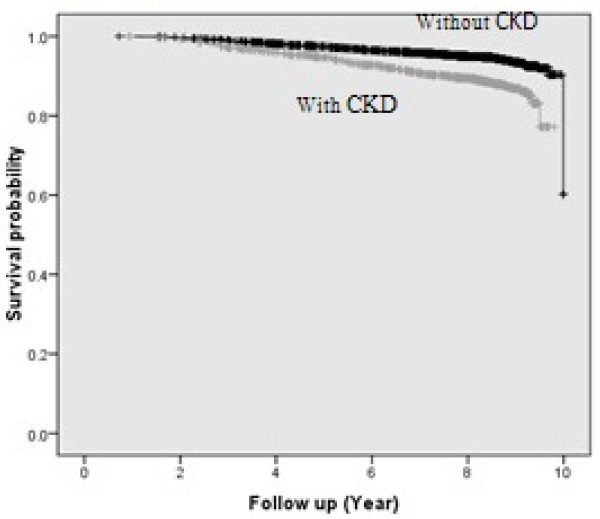
**Unadjusted cardiovascular survival (Kaplan-Meier) according to eGFR, estimated by MDRD formula [eGFR <60 mL/min per 1.73 m**^**2**^**was defined as chronic kidney disease (CKD)].** Log Rank test *X*^2^, 64.53: *P* < 0.001 HR: 2.18 (95% CI, 1.79-2.65)

There were 50,215 person-years of follow-up for the 6,209 study participants (43.1% male, mean age 47.4 years). The mean value of eGFR calculated using the MDRD formula in all the participants was 68.2 ml/min per 1.73 m^2^; overall, 22.2% (1381) had CKD, almost all of them (99%) were in stage 3a. Characteristics of the study population, stratified by CKD, are listed in Table 1. Most of cardiometabolic risk factors increased in subjects with CKD. Over a median (IQ 25–75) of 9.1 (8.7 - 9.6) years of follow up, there were 439 CVD events with an incidence rate of 87 per 10,000 person years. In fact, the 9.1-year unadjusted rate of freedom from CVD was significantly lower in subjects with CKD than in those without CKD (83.4%, vs 92.2% *P* <0.001; Figure 1).

**Table 1:** **Baseline characteristics of 6,209 study participants classified by CKD status**^**a**^

	**Overall (N = 6209)**	**Without CKD N = 4828**	**With CKD N = 1381**
			
**Age (years)** (12.3)	47.4	44.6 (11.2)	57.0 (11.2)
**Male n%** 43.1(2677)		47.3 (2286)	28.2 (390)
**Current Smoking n%** 14.2(884)		16.1 (778)	7.6 (105)
**Weight (Kg)** (12.3)	72.0	72.2 (12.3)	71.2 (11.5)
**BMI (Kg/m**^**2**^**)**(4.4)	27.6	27.4 (4.4)	28.5 (4.3)
**Normal weight n%** (1778)	28.6	31.1 (1501)	20 (276)
**Overweight n%** (2735)	44	43.7 (2112)	44.9 (620)
**Obese n%** (1702)	27.4	25.2 (1215)	35.1 (485)
**FPG (mg/dl)** (35.5)	101.0	98.7 (31.5)	109.0 (46.0)
**Diabetes Mellitus%(n)** 13.8(851)		11.4 (551)	21.6 (298)
**Total Cholesterol (mg/dl)** (45.4)	217.1	212.0 (43.5)	235.2 (47.2)
**Total Cholesterol ≥240 mg/dl%(n)** 27.8 (1725)		23.4 (1128)	43.2 (597)
**Triglycerides**^**b**^**(mg/dl)** 227)	158(109-	155 (106–222)	172 (123–242)
**Triglycerides ≥ 150 mg/dl%(n)** (3389)	54.5	52.8 (2547)	63.3(875)
**SBP (mmHg)** (19.9)	122.2	119.8 (18.2)	130.8 (22.9)
**DBP (mmHg)** (10.9)	79.3	78.7 (10.6)	81.4 (11.7)
**Hypertension(SBP/DBP ≥140/90 mmHg)%(n)**		23.3(1446)	19.5 (940) 36.4 (503)
**Serum creatinine, mg/dl** (0.2)	1.08	1.04 (0.1)	1.22 (0.3)
**e GFR (ml/min per 1.73 m**^**2**^**)**	68.2 (11)	72.2 (8.5)	53.9 (5.7)

Abbreviations: BMI, body mass; index FPG, fasting plasma glucose; CKD, chronic kidney disease; DBP, diastolic blood pressure; SBP, systolic blood pressure; HDL-C, high-density lipoprotein; GFR, glomerular filtration rate.

^a^ Data are given as mean (SD) value or percentage of participants unless otherwise specified.

^b^ Data are presented as median (25–75 inter quartile).

^c^ Calculated using MDRD formula [20].

Unadjusted hazard ratios (HR) for CVD events was 2.18 (95% CI 1.79-2.65) for subjects with CKD, compared to those without CKD. Moderate renal insufficiency still remained an independent predictor of CVD after adjustment for age and sex HR 1.25 (95% CI 1.00-1.55); yet in the final model it lost its significant association and the corresponding HR was 1.14 (95% CI 0.91-1.42) (Table 2). Details of multivariate-adjusted HRs for CVD events in normal-weight compared to the overweight and obese subjects with and without CKD are listed in Table 3. There was no significant interaction between BMI categories and CKD in development of CVD (*P* = 0.2).

**Table 2:** Hazard ratios for incident CVD in 6,209 study participants at baseline by CKD status

	**Without CKD**^**a**^**n = 4828**	**With CKD n = 1381**
**No. of person-years**	39539	10676
**No. of incidence CVD**	281	158
**Incidence rate**	71	148
**(per 10,000 person-years)**		
**Unadjusted hazard ratios**	1	2.18 (1.79-2.65)*
**Adjusted hazard ratios**		
**Model 1†**	1	1.25 (1.00-1.55)*
**Model 2‡**	1	1.14 (0.91-1.42)

^a^ CKD status based on MDRD category.

**†**Model 1 was adjusted for age, sex.

**‡**Model 2, we further adjusted for, body mass index, smoking (reference: no), history of diabetes mellitus (reference: no), Family history of premature CAD(reference: no), Hyper TG (reference: TG < 150 mg/dl), Hyper TC (reference: TC < 240), hypertension (reference: SBP/DBP < 140/90 mmHg).

*P < 0.05.

**Table 3:** **Hazard Ratios (HRs) for incident CVD in 6,209 participants at baseline by CKD**^**a**^**and BMI**^**b**^**categories**

	**Without CKD**^**a**^			**With CKD**		
**Variables**	**BMI 18.5-24.9 (N = 1398)**	**BMI 25–29.9 (N = 2314)**	**BMI ≥ 30 (N = 1548)**	**BMI 18.5-24.9 (N = 379)**	**BMI 25–29.9 (N = 418)**	**BMI ≥ 30 (N = 152)**
**No. of person-years**	12329	17274	9936	2094	4748	3834
**No. of incidence CVD**	64	135	82	30	78	50
**Incidence rate**	52	78	82	143	164	130
**(per 10,000 person-years)**						
**Unadjusted HRs**	1	1.52 (1.13-2.06)*	1.60 (1.15-2.23)*	2.94 (1.90-4.55)*	3.36 (2.41-4.67)*	2.64 (1.82-3.83)*
**Adjusted HRs**						
**Model 1†**	1	1.65 (1.22-2.22)*	2.03 (1.45-2.83)*	1.45(0.92-2.27)	2.02 (1.42-2.85)*	2.11 (1.42-3.12)*
**Model 2‡**	1	1.24 (0.86-1.79)	1.12 (0.62-1.99)	1.41(0.90-2.22)	1.43 (0.95-2.15)	1.08 (0.59-1.99)

^a^ CKD status based on MDRD category.

^b^ Body mass index.

**†**Model 1 was adjusted for age, sex.

**‡**Model 2, we further adjusted for, smoking (reference: no), history of diabetes mellitus (reference: no), family history of premature CAD (reference: no), Hyper TG (reference: TG < 150 mg/dl), Hyper TC (reference: TC < 240), hypertension (reference: SBP/DBP < 140/90 mmHg).

*P < 0.05.

## Discussion

This prospective community-based study, conducted on an urban population in Tehran, during almost 9.1 years of follow-up, shows that after adjustment for conventional CVD risk factors, moderate renal insufficiency (stage 3a: calculated by MDRD formula) is not an independent risk factor in predicting CVD events (HR:1.14 CI 95% 0.91-1.42), suggesting that the observed association of mild to moderate CKD with CVD events in the general population might be merely due to co-occurrence of the traditional CVD risk factors in this group. When participants with and without CKD were further classified by BMI categories, all the groups lost their statistically significant association with development of CVD events, indicating no significant interaction between CKD and BMI categories.

To the best of our knowledge this is the first study conducted on a cohort of a general population in the Middle East that investigated the role of CKD in developing CVD. Our findings support the conclusion of similar previous community-based studies that suggested the lack of an independent association of mild to moderate renal insufficiency with CVD outcomes. Results from the NHANES I during an average of 16.1 years follow-up of 2352 subjects in the U.S. general population, suggested the association between mild or moderate renal insufficiency and CVD is not independent, and appears to be due to the co-occurrence of traditional CVD risk factors with CKD [13]. Moreover as Culleton et al. [12] previously concluded in the Framingham study, after adjustment in the multivariable model for traditional CVD risk factors, mild CKD did not independently contribute to CVD risk in the general population. Also in a pooled analysis of community-based studies including the Atherosclerosis Risk in Communities Study, the Cardiovascular Health study, the Framingham Heart Study and the Framingham Offspring study, in the final adjusted model, CKD was not a significant predictor for outcomes such as myocardial infarction and fatal coronary heart disease (HR: 1.09 CI 95% 0.91-1.29) and for stroke (HR: 1.17 CI 95% 0.95-1.44); however, for the composite outcome after adding all-cause mortality to the previous CVD outcomes the association became significant (HR: 1.19 CI 95% 1.07-1.32) [22]. In contrast, there are community-based studies that demonstrated the independent association of moderate renal insufficiency with increased risk of CVD events and mortality in general population [7,9,22-28]. GO et al. [29] after 2.84 years of follow-up of 1,120,295 participants, reported an independent, graded association between a reduced eGFR and the risk of cardiovascular events, in a large, community-based population. However, as listed in the possible limitations of this study, the authors did not have information to adjust for unmeasured confounders such as BMI and level of blood pressure. Moreover, in our analyses across different BMI categories, we observed the loss of significance after further adjustment in all categories of BMI indicating no interaction between BMI and CKD (*P* =0.2). However, we have to be cautious when interpreting the HR in overweight participants with CKD; this seems to be marginally significant (HR: 1.43 95% CI, 0.95-2.15). Given the phenomenon of the “obesity paradox” reported in patients with advanced stages of CKD [5], our results showed that the “obesity paradox” cannot be regarded as a major concern in a general population. However, further investigations are needed to clarify the issue.

The main mechanism that has been proposed to explain the increased risk of CVD in CKD is coexistence of traditional and nontraditional cardiometabolic risk factors with CKD [30]. Accordingly, we observed higher prevalence of traditional risk factors in subjects with CKD as compared to subjects without CKD. Moreover, some other investigators suggested that CKD may be a marker merely for existence of clinically silent atherosclerotic disease or a marker of duration and severity of baseline cardiometabolic risk factors [13].On the other hand ongoing research suggests the possible causal mechanisms and presence of non-traditional CVD risk factors in CKD populations that contribute to a higher prevalence of CVD and accelerated atherosclerosis in renal insufficiency [31,32].

Our study has both limitations and strengths that deserve comment. We know that use of various indirect estimates of renal function might lead to different conclusions and should be interpreted with caution [33]. In this study, like most of the similar epidemiologic studies, we used the MDRD equation formula to estimate GFR. MDRD formula was derived from a population predominantly with CKD and the efficient performance of this equation in general population is open to question [34]. Furthermore MDRD has been shown to underestimate true GFR in healthy populations [35]. The formula has not been validated in our population and differences in general characteristics of our subjects to those originally used to MDRD formula could influence the accuracy of our classifications. Also as another measurement limitation, it must be addressed that data regarding urinary albumin and protein excretion were not collected, and hence the risk reported by some studies emphasizing the notable role of albuminuria (stages 1 and 2 CKD) could not be assessed for possible CVD outcomes in our population [36-38]. Misclassification could have occurred in this study, as subjects could have had changes in their BMI and modifications of other relevant cardiovascular risk factors during the follow-up period. However, this concern is true for most epidemiologic studies that use only baseline information for the exposure variable. The physiologic decline of GFR by aging might also be a potential source of misclassification and considering the single measurement, some healthy individuals might have been misclassified as having CKD, moreover due to possible sampling error by single measurement, some with an acute rise in GFR might have been misclassified with respect to their CKD status, although this may be unlikely in a community-based study. Finally this study might not be statistically powered enough to show the possible sex stratified association and the interaction between BMI and mild to moderate CKD in developing CVD. On the other hand, the strengths of our analysis include the examination of a large community-based sample of men and women across a broad age spectrum and the standardized assessment of CVD risk factors. In addition, we were able to adjust for several important confounding variables to explore the effects of BMI groups and CKD status in CVD events. Moreover, only about 11% (777 of 6,992) of the baseline participants were lost to follow-up, which indicates low attrition rate.

## Conclusions

In conclusion our findings imply that mild to moderate CKD is not an independent risk factor for developing cardiovascular disease in a general population, and no significant interaction was found between BMI and CKD categories in CVD outcome. Considering the co-existence of CKD and cardiometabolic risk factors, more attention must be given to the appropriate management of these risk factors to reduce CVD events.

## Abbreviations

CVD, Cardiovascular disease; CKD, Chronic kidney disease; GFR, Glomerular filtration rate; eGFR, Estimated glomerular filtration rate; ESRD, End-stage renal disease; BMI, Body mass index; TLGS, Tehran Lipid and Glucose Study; TC, Total cholesterol; TG, Triglyceride; ADA, American Diabetes Association; CAD, Coronary artery disease; NKF, National Kidney Foundation; MDRD, Modification of Diet in Renal Disease; SBP, Systolic blood pressure; DBP, Diastolic blood pressure.

## Competing interests

All authors declare that they have no competing interests.

## Authors’ contributions

FH conceived of the study and participated in its design, advised throughout the study and its final approval and helped to draft the manuscript. MB and HAG participated in its design and coordination, drafted the manuscript and performed the statistical analysis. AAN, FS and FA reviewed the study design and revised the manuscript. All authors read and approved the final manuscript.

## Pre-publication history

The pre-publication history for this paper can be accessed here:

http://www.biomedcentral.com/1471-2369/13/59/prepub
